# Anterolateral Thigh Flap for Abdominal Wall Defect After Debridement for Meleney's With Fournier's Gangrene: A Case Report of a Rare Outcome

**DOI:** 10.7759/cureus.72906

**Published:** 2024-11-02

**Authors:** Arunkumar Lakshmanan, Gokulesh DG, Sabari Girieasen M, Kalpana Devi AK

**Affiliations:** 1 Institute of General Surgery, Madras Medical College, Chennai, IND

**Keywords:** anterolateral thigh flap, fournier’s gangrene, meleney’s gangrene, split-thickness skin grafting, wound debridement

## Abstract

Meleney's gangrene is a synergistic polymicrobial infection of the anterior abdominal wall causing rapidly progressive necrotizing fasciitis of skin and subcutaneous tissues. When combined with Fournier's gangrene, the mortality rates are higher. Here, we discuss a case of Meleney’s with Fournier’s gangrene managed with appropriate antibiotics and extensive wound debridement, followed by a successful split-thickness skin grafting of the lower anterior abdominal wall and scrotum. The patient presented after three months with a ventral hernia. The hernial sac was de-epithelialized and preperitoneal on-lay mesh repair was done. The defect necessitated a flap cover. Hence, a lateral circumflex artery-based pedicled anterolateral thigh (ALT) flap was raised and placed over the mesh to restore the fascial contour and structural integrity of the abdominal wall.

## Introduction

Fournier's gangrene and Meleney's gangrene are rapidly progressive bacterial gangrene. The production of tissue-destructive enzymes, collagenases, and endotoxins as a result of synergism causes obliterative endarteritis of subcutaneous blood vessels. This leads to tissue necrosis and aids the rapid spread of infection along tissue planes [1]. Hence, these conditions require urgent surgical and medical intervention as most of the time patients present with septic shock [2]. After a course of appropriate antibiotics and wound debridement, the infection resolves and the wound gets covered by healthy granulation tissue that requires a skin cover. This can be addressed with split-thickness skin grafting in the majority of the cases. However, other expected complications require further innovative management as in our case described below.

## Case presentation

**Figure 1:**
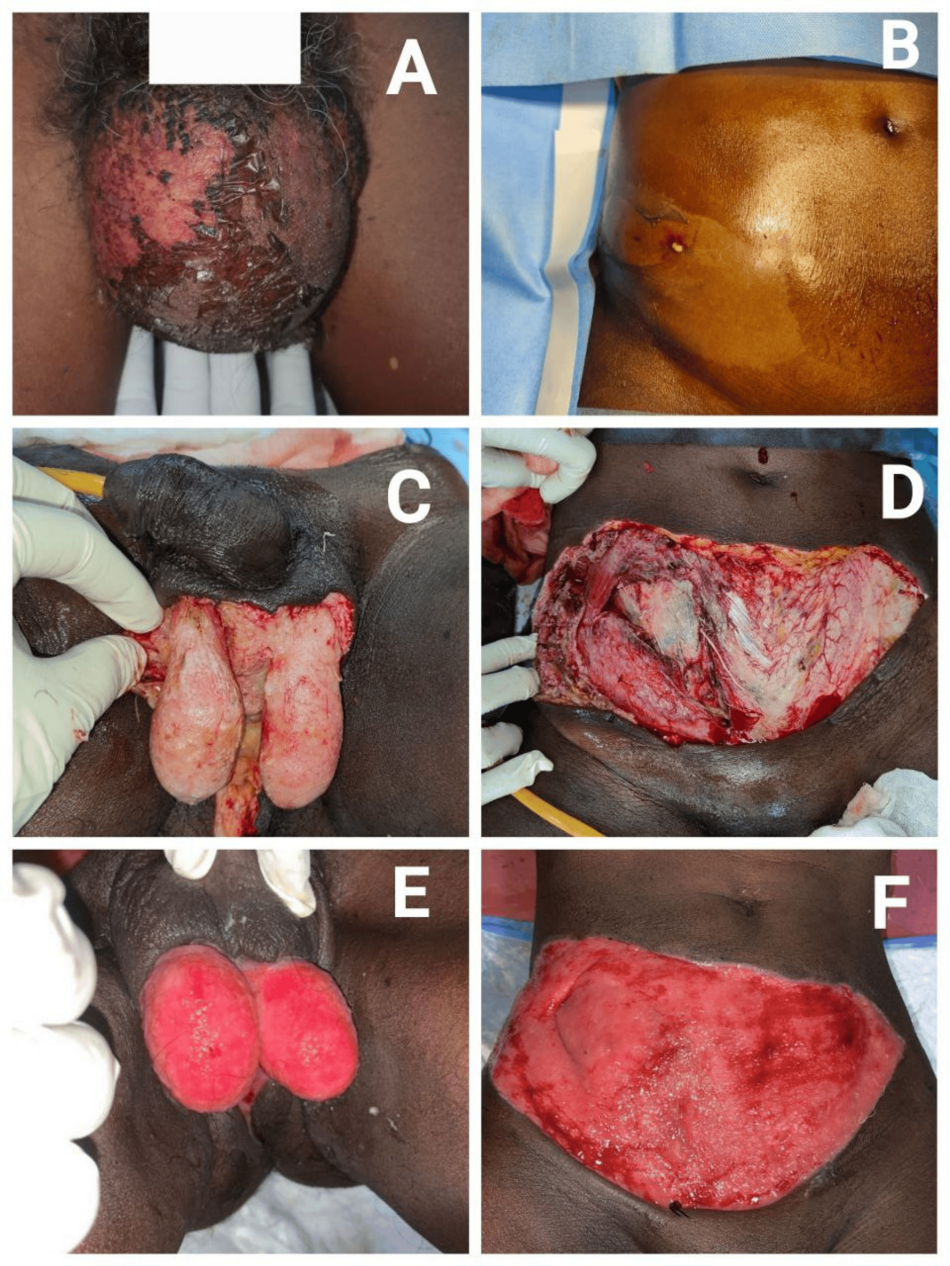
Initial presentation of the patient and after successive wound debridement and dressing. A and B: Discoloration and skin changes in the scrotum and abdominal wall, respectively. C and D: Initial wound debridement showing pale unhealthy tissues (notice the sloughed-out abdominal muscles). E and F: Healthy red granulation tissue after serial debridement and dressing.

A 45-year-old male was admitted with complaints of scrotal swelling, blackish discolouration, and peeling of skin over the scrotum for the past one week. He also had similar complaints over the lower abdomen for three days. On examination, the skin was stretched, shiny and desquamated over both regions. There was an additional pus point over the right lower abdominal wall. Warmth, tenderness, and palpable crepitus were present over both regions. Blood investigations showed an elevated total leukocyte count and an ultrasonogram (USG) revealed multiple air pockets in the subcutaneous plane of the scrotum tracking up to the right inguinal region. Diagnosis of Fournier’s gangrene with synergistic Meleney’s gangrene was made. We proceeded with extensive debridement of the necrotic tissues in the scrotum and lower anterior abdominal wall. The external and internal oblique muscles were found to be sloughed off on the right side while the abdominal musculature on the opposite side was healthy. The patient received regular bedside wound debridement and dressing along with culture-sensitive antibiotics, which aided the formation of healthy granulation tissue (Figure 1).

**Figure 2:**
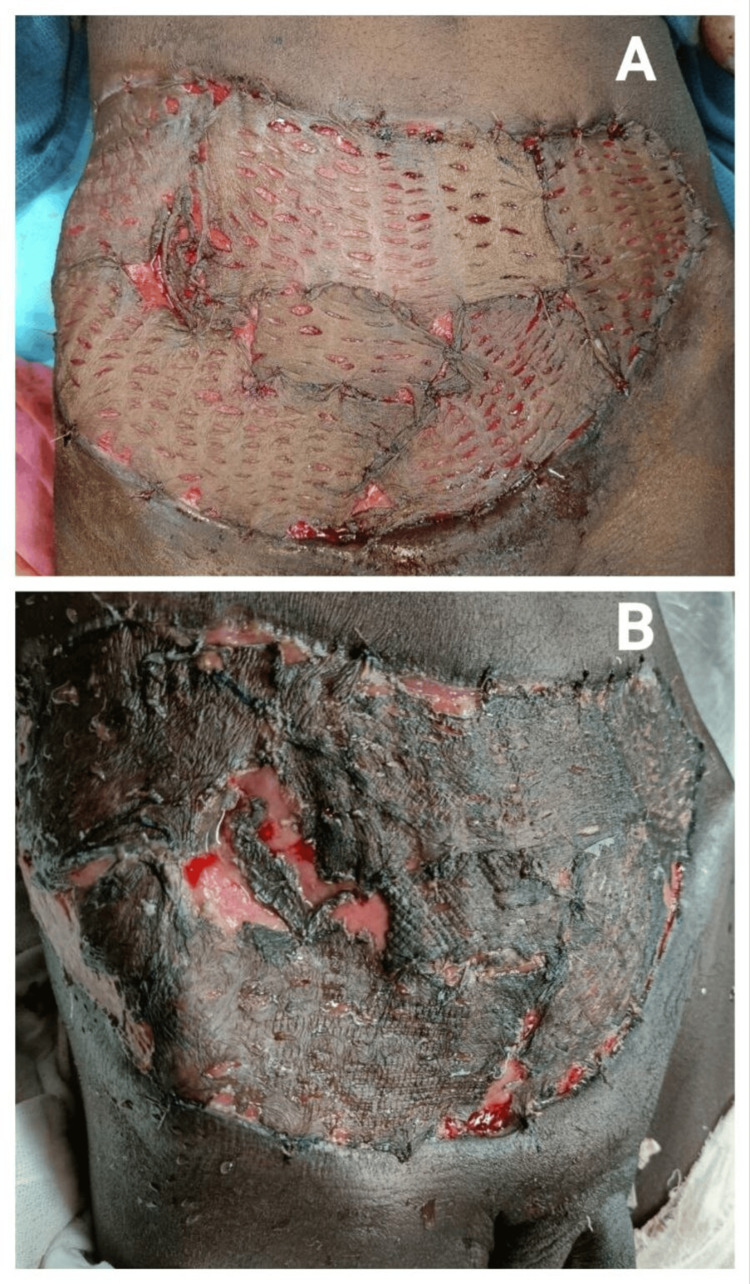
A: Split-thickness skin grafting on the abdominal wall. B: After successful graft uptake.

A split-thickness skin graft was placed on the scrotum and anterior abdominal wall after two weeks. He was discharged with a graft uptake of >90% and was on regular follow-up (Figure 2).

**Figure 3:**
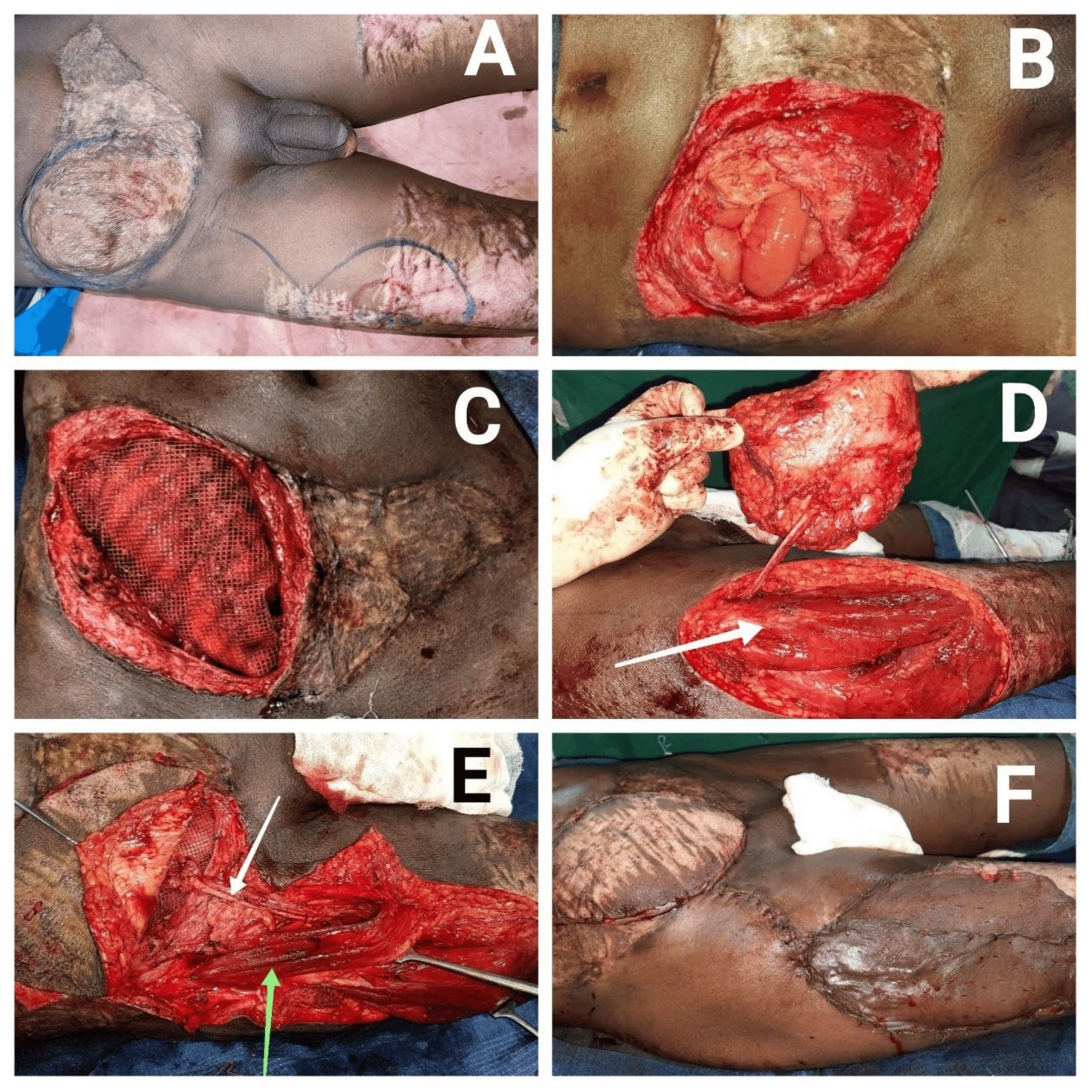
Operative images. A: Preoperative planning of the ALT flap. B: De-epithelialization and opening of hernial sac followed by reduction of contents. C: Pre-peritoneal onlay mesh placement. D: Elevation of ALT flap (white arrow pointing to the rectus femoris). E: Tunnelling the pedicled flap (white arrow) under the sartorius muscle (green arrow) and along the subcutaneous canal to the defect. F: Fixation of the flap to the defect and covering of the donor area with a skin graft. ALT: anterolateral thigh.

The risk of ventral hernia was explained due to a lack of abdominal muscles on the right. He presented three months later with a cough impulse in the right lower abdomen (Video 1) and a computed tomography (CT) scan revealed a right lower anterior abdominal wall defect. The patient was planned for a preperitoneal onlay mesh repair with a bailout plan of intraperitoneal mesh if the sac could not be de-epithelialized. However, the hernial sac was de-epithelialized, and contents were reduced, followed by a preperitoneal mesh placement. The 10 x 8 cm defect also necessitated a flap to cover the mesh and strengthen the abdominal wall. Hence, an anterolateral thigh (ALT) flap based on the lateral circumflex femoral artery was elevated and transposed under the sartorius muscle to cover the defect. The donor site was covered using a split-thickness skin graft from the contralateral thigh (Figure 3).

**Video 1:** Visible cough impulse three months after split-thickness skin grafting.

The patient was discharged after 14 days with a healthy flap and the patient was reviewed weekly for four weeks and monthly for three subsequent months. The patient was advised an abdominal binder and gradual weight lifting only after six weeks. Cough impulse was absent at the three-month follow-up (Video 2).

**Video 2:** Post-operative video showing restored abdominal contour during follow-up (note the absence of cough impulse).

## Discussion

Fournier’s gangrene is a necrotizing fasciitis affecting perineal, scrotal, and genital regions often associated with general sepsis and a 40% mortality rate [2]. The rapid spread of infection along the Dartos, Colles, and Scarpa's fascias allows early involvement of the abdominal wall [3]. Synergistic polymicrobial aerobic and anaerobic bacteria are implicated, with group A *Streptococcus*, *Staphylococcus*, *Escherichia coli*, and *Pseudomonas aeruginosa* being the commonest [4]. Meleney's gangrene is also a life-threatening necrotizing fasciitis of the anterior abdominal wall frequently associated with immunocompromised states like active corticosteroid usage, post-surgery, diabetes mellitus, and HIV [5]. Starting as a superficial skin infection, it spreads rapidly into the subcutaneous plane and causes tiny vessel thrombosis leading to necrosis and gangrene later on. It is a synergistic bacterial gangrene due to non-hemolytic *Streptococcus* and hemolytic *Staphylococcus* [5].

These disease processes are surgical emergencies that require medical resuscitation in addition, as the patient often presents with septic shock. Initiation of empirical broad-spectrum antibiotics covering gram-positive, gram-negative, and anaerobic organisms is imperative, but early aggressive surgical exploration and debridement are vital [2,6]. The first debridement must be early and extensive, followed by regular bedside wound debridement. This is crucial to remove the septic foci [2]. Primary closure or delayed primary closure, healing by secondary intention, skin grafting, tissue expansion, and free or pedicled flaps are the traditional options for wound closure after debridement [6]. Skin grafting or local tissue rearrangements achieve wound closure in most cases, as was true in our case. However, ventral hernia was an expected complication because external and internal oblique muscles were lost during serial debridement. Since identification of the defect margin is crucial for planning further treatment, expectant management was done till the defect margin came into form.

**Figure 4:**
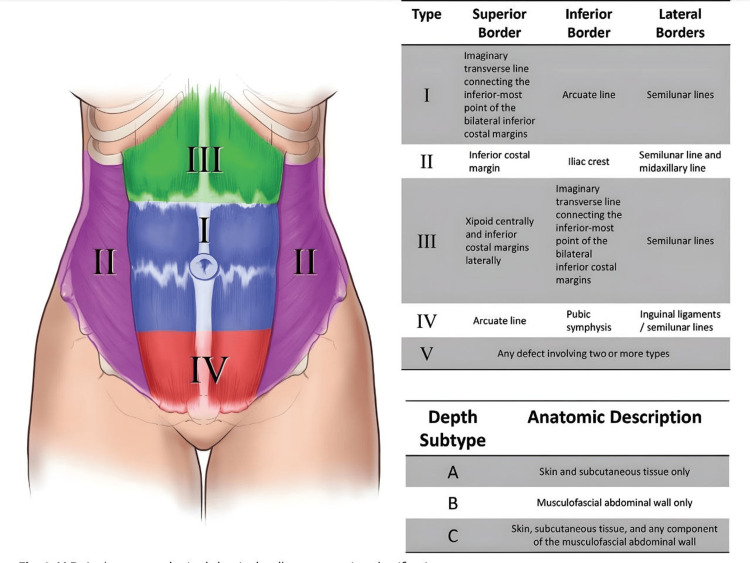
MD Anderson oncologic abdominal wall reconstruction classification system. Source: Mericli et al. [7]. Used with permission from Wolters Kluwer Health, Inc. (License No.: 5895340980810, CC-BY-NC).

MD Anderson's abdominal wall reconstruction classification was used to quantify the defect and decide upon the management [7]. This system divides the abdominal wall into four surface area types (I-IV) and three depth subtypes (A, B, C). Type I defect area lies between an imaginary transverse line joining the inferior-most point of the inferior costal margins superiorly, arcuate line inferiorly, and semilunar lines laterally. The type I defect area is flanked by type II defect areas laterally, type III defect area superiorly, and type IV defect area inferiorly. Type V defect area involves two or more types (Figure 4).

Depth subtype A defects involve only skin and subcutaneous tissue while subtype B involves only the musculofascial abdominal wall. Type C defects are defined by loss of skin, subcutaneous tissue, and any component of the musculofascial abdominal wall. Our patient had a type V surface area defect (involving type IV (major) and II areas) with depth subtype C. Hence, a mesh placement with a fasciocutaneous flap cover was decided. According to Mericli et al., the mainstay for the reconstruction of type IV defect is with pedicled flaps based on the descending branch of the lateral femoral circumflex vessels [7]. Hence an ALT flap was planned.

The ALT flap was first described in 1984 by Song et al., who outlined its fasciocutaneous nature based on perforators of the descending branch of the lateral circumflex femoral artery [8]. There are multiple advantages to using this flap. It can be taken as a free flap or pedicled flap. The long vascular pedicle provides a wide arc of rotation. It is crucial to tunnel the flap proximally beneath the rectus femoris muscle or under the sartorius while utilizing a pedicled ALT flap to lengthen the pedicle and make a large subcutaneous tunnel into the belly without squeezing the pedicle. Also, branches to the rectus femoris and tensor fascia lata can be divided to achieve extra pedicle length. The skin size and pedicle length determine the maximum cranial reach of the flap [9].

The flap can be customized in both size and thickness to suit the patient's specific needs, and its ability to be harvested with or without fascia adds to its versatility. It can be harvested as a cutaneous, fasciocutaneous, or myocutaneous flap. The inclusion of the lateral femoral cutaneous nerve can make the flap sensate [10]. Another key advantage of the flap is the ability to harvest significant amounts of tissue without functional impairment of the donor site [11]. Additionally, the flap provides a good cosmetic outcome, with the donor site on the thigh being easily concealed under clothing [8]. The robust vascularity of the flap ensures rapid healing, even in the presence of prior infection, and the flap’s large size allows for coverage of extensive areas with minimal risk of failure [12]. However, ALT flap harvest can be technically demanding in patients with thick subcutaneous fat, such as those who are obese. In such cases, modification of the flap design may be necessary to ensure an adequate blood supply. Another limitation of the flap is its inability to provide a bony component [9]. Further research into long-term outcomes and refinements in surgical technique will continue to enhance the role of the ALT flap in abdominal wall reconstruction.

## Conclusions

This case report details the success story, not of a rare case, but a common case with a rare outcome. Meleney’s with Fournier’s gangrene has a very high mortality rate. Effective, extensive, and timely wound debridement helped in the removal of the septic foci and pushed the patient into recovery. The combined efforts of regular dressing, antibiotics, and nutrition helped in wound healing, allowing for further reconstructive options.

The versatility, large size, reliable vascularity, and minimal donor site morbidity make the ALT flap ideal for complex reconstructions. Even though the flap needs modification in obese patients, with proper preoperative planning and meticulous surgical technique, the flap can provide durable, functional, and aesthetically pleasing outcomes for suitable patients.
